# National travel distances for emergency care

**DOI:** 10.1186/s12913-022-07743-7

**Published:** 2022-03-24

**Authors:** Anagha Tolpadi, Marc N. Elliott, Daniel Waxman, Kirsten Becker, Elizabeth Flow-Delwiche, William G. Lehrman, Debra Stark, Layla Parast

**Affiliations:** 1 RAND Corporation, 1776 Main Street, Santa Monica, CA 90407 USA; 2 Centers for Medicare & Medicaid Services, Baltimore, MD 21244 USA

**Keywords:** Emergency department, Access to care, Travel time, Travel distance, Geographic distance

## Abstract

**Background:**

Most emergency department (ED) patients arrive by their own transport and, for various reasons, may not choose the nearest ED. How far patients travel for ED treatment may reflect both patients’ access to care and severity of illness. In this study, we aimed to examine the travel distance and travel time between a patient’s home and ED they visited and investigate how these distances/times vary by patient and hospital characteristics.

**Methods:**

We randomly sampled and collected data from 14,812 patients discharged to the community (DTC) between January and March 2016 from 50 hospital-based EDs nationwide. We geocoded and calculated the distance and travel time between patient and hospital-based ED addresses, examined the travel distances/ times between patients’ home and the ED they visited, and used mixed-effects regression models to investigate how these distances/times vary by patient and hospital characteristics.

**Results:**

Patients travelled an average of 8.0 (SD = 10.9) miles and 17.3 (SD = 18.0) driving minutes to the ED. Patients travelled significantly farther to avoid EDs in lower performing hospitals (*p* < 0.01) and in the West (*p* < 0.05) and Midwest (*p* < 0.05). Patients travelled farther when visiting EDs in rural areas. Younger patients travelled farther than older patients.

**Conclusions:**

Understanding how far patients are willing to travel is indicative of whether patient populations have adequate access to ED services. By showing that patients travel farther to avoid a low-performing hospital, we provide evidence that DTC patients likely do exercise some choice among EDs, indicating some market incentives for higher-quality care, even for some ED admissions. Understanding these issues will help policymakers better define access to ED care and assist in directing quality improvement efforts. To our knowledge, our study is the most comprehensive nationwide characterization of patient travel for ED treatment to date.

**Supplementary Information:**

The online version contains supplementary material available at 10.1186/s12913-022-07743-7.

## Background

How far patients travel for emergency department (ED) treatment may reflect both patients’ access to care and severity of illness. While patients who call for an ambulance for emergency medical services usually are taken to the nearest ED with appropriate services, most patients’ visits to an ED are not due to an accident or injury, and most arrive by their own transport [1]. Depending on a patient’s location and/or type of care needed, a patient may not choose the nearest ED. Understanding the distance patients travel to visit EDs is important in understanding ED utilization patterns, access to care, and the effect of quality information.

Prior studies of geography and EDs typically consider: (1) actual patterns of ED utilization, mapping the travel distance/time between a patient’s home (or ambulance pickup location) and the ED, or (2) the availability of ED services for populations, usually by matching census data to hospital locations. Such studies have estimated travel distances/times to the EDs among patients within limited contexts: in specific regions, hospital chains, subgroup of patients, or by matching census location data to hospital locations [2–6]. For example, one study of Medicaid-insured adults at two large hospital chains found the average travel time was between 15.8 and 17.9 min [3]. Another study using Indiana Public Health Emergency Surveillance data found that the travel distances to an ED were < 5 miles for 60% of ED visits, 5–20 miles for 30%, and > 20 miles for 8% [4]. A study using 2013 Census and the national ED inventories data found that 71% of the US population has access to an ED within 30 min, and 98% has access within 60 min, with more limited access to teaching hospitals and lower access in rural states [6].

In contrast, we provide the most comprehensive nationwide characterization of how far people travel to visit an ED by examining the travel time and distance between a patient’s actual home address and the ED visited using geocoded data from patients (regardless of insurance status) who visited 50 hospital-based EDs of varying size and rurality across the US. We also investigate how these distances/times vary by patient and hospital characteristics.

## Methods

### Study design and participant/hospital selection

We drew a random sample of eligible adult ED patients discharged to home between January and March 2016 from their visit to one of 50 national hospital-based EDs.

These 50 hospitals were a random proportionate stratified sample from categories based on size and region of all non-specialty hospitals with 14,000+ annual ED visits. Patient eligibility criteria were the same as those for the Hospital Consumer Assessment of Healthcare Providers and Systems (HCAHPS) Survey [7] except: ED patients admitted to the hospital following the ED visit were ineligible. Sampled patients were given the Emergency Department Patient Experience of Care (EDPEC) Discharged to Community (DTC) Survey (which became the ED CAHPS® [Consumer Assessment of Healthcare Providers and Systems] Survey in March 2020). This study was approved by the Institutional Review Board at the study team’s institution. Additional details regarding study design and sampling, hospital and patient selection, and the survey instrument appear in the Additional file 1.

### Analytic sample and geocoding process

Of the 16,006 sampled ED DTC patients, 4.0% were excluded due to incomplete addresses (*n* = 52), out-of-scope addresses (U.S. Territories or non-US) (*n* = 3), and because they were determined to be ineligible for the study after sampling (*n* = 585).

Patient and hospital addresses were geocoded using ArcGIS [8]. We calculated the Euclidean distance (in miles) and driving time based on the quickest route (in minutes) between patients’ home address and the hospital-based ED. Additional details appear in the Additional file 1.

We excluded 554 patients whose driving time to the hospital exceeded 2 h, as such patients likely did not travel from the home address in their hospital’s administrative record. After exclusions, our analytic sample had 14,812 cases.

### Hospital and patient characteristics

The independent variables were distance and driving time between patients’ home address and the hospital-based ED. Hospital and patient characteristics (described below) were used to examine differences by distance/time.

Hospital characteristics, including teaching affiliation, ED volume (categorized using number of annual ED visits), and rurality, came from the 2015 American Hospital Association (AHA) database [9]. ED volume (number of annual ED visits) was categorized as low (14,000-24,999), medium (25,000-49,999), or high (50,000+). Census region was coded using the hospital’s address. The HCAHPS Summary Star Rating, which combines the star ratings of 10 patient experience measures (including the extent to which patients recommend the hospital), for patients discharged from January 2016 through December 2016 came from the Provider Data Catalog [10]; “low-performing” hospitals were defined as those with HCAHPS Summary Star Ratings of 2 or lower on a 1 to 5 scale. All patient characteristics came from hospital administrative data.

### Statistical analyses

Summary statistics of the distance and driving time between a patient’s home address and the hospital-based ED were calculated overall and by hospital and patient characteristics. We ran mixed-effects regression models predicting distance and driving time outcomes, with fixed effects for hospital and patient characteristics and random hospital effects to explore whether the two outcomes (distance and driving time) varied by hospital or patient characteristics. Missing patient characteristics were imputed using the hospital-level mean.

Statistical analyses were conducted using SAS 9.4 and figures were created using R 4.0.3.

## Results

Table 1 compares the characteristics of the 50 participating hospitals to all non-specialty hospitals with an ED. Most participating hospitals were urban (90%) and had high ED volume (66%); the hospitals reflected a mix of teaching statuses (26% major, 48% minor, and 26% non-teaching hospitals, respectively) and census regions.

**Table 1:** Hospital Characteristics of Emergency Departments Nationally and in Study

Hospital Characteristic	National (*N* = 4436) *N* (%)	In Study (*N* = 50) *N* (%)
Rurality		
Rural	1866 (42%)	5 (10%)
Urban	2570 (58%)	45 (90%)
Teaching Affiliation		
Major	238 (5%)	13 (26%)
Minor	1403 (32%)	24 (48%)
Non-Teaching	2795 (63%)	13 (26%)
Census Region		
Midwest	1335 (30%)	11 (22%)
Northeast	536 (12%)	10 (20%)
South	1642 (37%)	20 (40%)
West	872 (20%)	9 (18%)
Other^a^	51 (1%)	0 (0%)
ED Volume^b^		
Low	688 (16%)	1 (2%)
Medium	1032 (23%)	16 (32%)
High	953 (21%)	33 (66%)
Low Performing Hospitals^c^		
Yes	574 (17%)	12 (24%)
No	2733 (83%)	38 (76%)

Note: National hospitals reflect all hospitals in the 2015 AHA database that are not listed as being specialty hospitals or not having an emergency department; Percentages are calculated excluding missing values

^a^Other includes hospitals in PR, GU, VI, MP, and AS

^b^ED volume was classified using number of annual ED visits: Low: 14,000-24,999; Medium: 25,000-49,999; High: 50,000+. 1763 (40%) national hospitals had < 14,000 annual ED visits and thus were ineligible for this study based on size

^c^Low performing hospitals are classified as those with HCAHPS Summary Star Ratings of 2 or lower

The majority of patients were women (59.3%), with 15.2% aged 18–24 and 3.5% aged 85+ (Table 2), similar to the US adult ED DTC population (58.6% women, 16.3% aged 18–24, and 3.2% aged 85+) [11].

**Table 2:** Travel Distances/Times from a Patient’s Home to the Emergency Department by Hospital and Patient Characteristics

	Miles to Hospital		Driving Minutes to Hospital	
	Mean (SD)	Coefficient (95% CI)	Mean (SD)	Coefficient (95% CI)
**Hospital Characteristics**				
Rurality				
Rural (*N* = 1494)	11.08 (12.49)	2.68 (− 0.13, 5.48)	25.85 (25.51)	8.19 (2.70, 13.69)**
Urban [ref] (*N* = 13,318)	7.75 (10.69)	0.00	16.38 (16.67)	0.00
Teaching Affiliation				
Major [ref] (*N* = 3852)	10.15 (13.06)	0.00	20.62 (20.28)	0.00
Minor (*N* = 7165)	6.61 (9.81)	−2.95 (−5.07, − 0.83)**	14.22 (15.91)	− 5.45 (−9.59, − 1.30)**
Non-Teaching (*N* = 3795)	8.78 (10.14)	− 2.12 (−4.55, 0.31)	19.87 (18.23)	−3.30 (−8.05, 1.45)
Census Region				
Midwest (*N* = 3277)	7.87 (11.24)	−2.13 (−4.29, 0.02)	16.48 (17.47)	−5.11 (−9.33, − 0.89)*
Northeast (*N* = 2945)	7.29 (9.11)	−1.52 (−3.73, 0.68)	16.91 (17.35)	− 1.73 (−6.05, 2.58)
South [ref] (*N* = 5859)	9.25 (11.72)	0.00	19.73 (19.57)	0.00
West (*N* = 2731)	6.71 (10.33)	−2.11 (−4.54, 0.32)	13.69 (14.73)	−4.80 (−9.56, − 0.04)*
ED Volume^a^				
Low (*N* = 267)	9.14 (13.48)	2.66 (−3.40, 8.72)	18.07 (14.42)	5.06 (−6.79, 16.91)
Medium (*N* = 4624)	8.09 (10.68)	0.15 (−1.73, 2.03)	18.27 (18.73)	1.38 (−2.31, 5.06)
High [ref] (*N* = 9921)	8.06 (10.97)	0.00	16.87 (17.71)	0.00
Low Performing Hospitals^b^				
Yes (*N* = 3595)	5.54 (9.21)	−2.75 (−4.78, −0.72)**	11.92 (13.37)	−5.69 (−9.67, −1.71)**
No [ref] (*N* = 11,217)	8.91 (11.31)	0.00	19.07 (18.91)	0.00
**Patient Characteristics**				
Age				
18–24 [ref] (*N* = 2246)	8.81 (12.75)	0.00	17.91 (19.58)	0.00
25–34 (*N* = 3243)	8.04 (10.85)	−0.61 (−1.17, − 0.05)*	17.01 (17.50)	− 0.62 (− 1.52, 0.27)
35–44 (*N* = 2467)	7.99 (10.37)	−0.64 (− 1.23, − 0.04)*	17.20 (17.34)	−0.53 (− 1.48, 0.42)
45–54 (*N* = 2360)	8.26 (10.69)	− 0.50 (− 1.10, 0.10)	17.85 (18.27)	−0.19 (− 1.15, 0.77)
55–64 (*N* = 1801)	8.12 (10.92)	− 0.49 (− 1.14, 0.16)	17.77 (18.51)	0.01 (− 1.02, 1.05)
65–74 (*N* = 1298)	8.25 (10.82)	− 0.60 (− 1.32, 0.11)	18.01 (18.12)	−0.41 (− 1.55, 0.73)
75–84 (*N* = 839)	7.53 (9.80)	−1.04 (− 1.87, − 0.21)*	16.87 (17.19)	−1.00 (− 2.32, 0.33)
85+ (*N* = 514)	5.37 (7.93)	−3.03 (−4.03, − 2.02)***	12.61 (13.75)	−4.82 (−6.42, − 3.22)***
Sex				
Female [ref] (*N* = 8782)	8.06 (10.88)	0.00	17.29 (17.74)	0.00
Male (*N* = 6029)	8.13 (11.01)	0.02 (−0.33, 0.36)	17.40 (18.35)	0.03 (−0.52, 0.57)

Note: Coefficient, confidence intervals and significance are derived from multivariate models for each outcome (driving distance or time) with fixed effects for hospital and patient characteristics and random hospital effects. Random effects for hospitals were included to control for differences between hospitals. Missing patient characteristics were imputed using the hospital-level mean in models. Table 2 shows the coefficients, confidence intervals and characteristics associated with hospital and patient characteristics; Missing categories not shown

*Abbreviations*: *ED* emergency department, *ref* reference category, *SD* standard deviation, *CI* confidence interval

^a^ED volume was classified using number of annual ED visits: Low: 14,000-24,999; Medium: 25,000-49,999; High: 50,000+

^b^Low performing hospitals are classified as those with HCAHPS Summary Star Ratings of 2 or lower

^*^p < 0.05

^**^p < 0.01

^***^p < 0.001

**Fig. 1:**
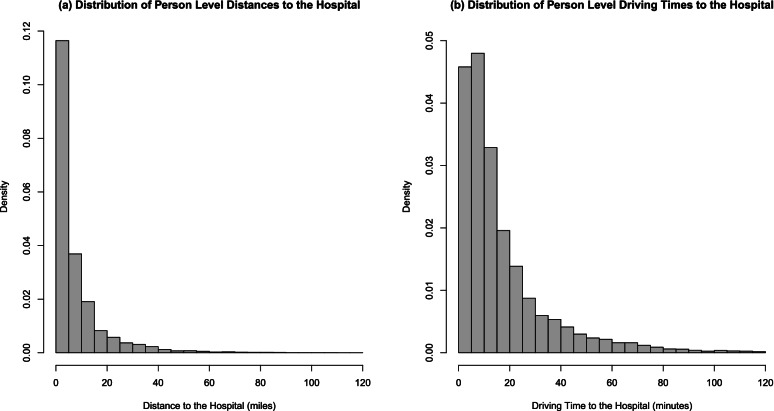
Distributions of (**a**) Distance and (**b**) Driving Time from Patient’s Home to Hospital

Distance and driving time from a patient’s home to hospital were highly correlated (r = 0.94). The distributions of these distances/times were right-skewed (Fig. 1), with most patients living close to the hospital they visited. Patients travelled an average of 8.0 (SD = 10.9) miles (median of 4 miles), with an interquartile range of 2–10 miles, and travelled an average of 17.3 (SD = 18.0) driving minutes (median of 11 min), with an interquartile range of 6–22 min.

Travel distance/time varied by hospital and patient characteristics (Table 2). Patients travelled significantly less far to visit EDs in lower performing hospitals (by 6 min, *p* < 0.01), in hospitals in the West (by 5 min, *p* < 0.05) and Midwest (by 5 min, *p* < 0.05), and in minor teaching hospitals (by 5 min compared to major teaching hospitals, *p* < 0.01). Patients travelled significantly farther to hospitals in rural areas (by 8 min, *p* < 0.01). Patients aged 18–24 travelled longer distances than patients aged 25–44 and 75+ and had significantly (*p* < 0.001) longer driving times than patients aged 85+ (by 5 min).

## Discussion

Our study is the most comprehensive nationwide characterization of patient travel for ED treatment and extends the findings of smaller-scale or more specialized studies by identifying new determinants of ED travel distances/times.

We found that patients travelled 6 min farther to avoid EDs in low-performing hospitals (with 2-star quality ratings or less) (*p* < 0.01), which perhaps indicates that patients will travel farther to avoid EDs that they perceive as worse. Patients aged 18–24 similarly travelled about 5 min farther than patients aged 85+. As expected, we found that patients travelled significantly farther to EDs in rural areas, where the distance between EDs is likely longer.

### Limitations

Our study has several limitations. Our sampling frame was restricted to DTC patients. Our findings, therefore, may not be generalizable to the 10.4% of ED visits [12] where patients are admitted to the hospital from the ED. The travel patterns for DTC patients, however, may better represent cases in which choice plays a meaningful role. While the 50 participating hospitals were selected to try to achieve national representation in size and census region, hospitals that participated in this study voluntarily agreed to participate, and we restricted to hospitals with 14,000+ annual ED visits. As such, while this study is nationally representative of hospitals with 14,000+ annual ED visits (where 92% of annual ED visits take place), it is not representative of all hospitals. Furthermore, we did not know whether patients arrived from home. While we attempted to address this by excluding patients with travel times above two-hours, it is likely that we included some patient addresses that do not reflect their specific location prior to visiting the ED. Additionally, we did not have information available about whether a patient was diverted to an ED that was not necessarily one of their choosing. Future work on patient choice and diversion for various reasons would further contribute to this research. Lastly, there are several potentially important variables that were unavailable (e.g., arrival by ambulance, insurance status, severity of illness/acuity of condition, diagnosis, race/ethnicity, socioeconomic status, and availability of primary care providers); results may differ if these variables are confounders. Future work is needed to see if and how results vary adjusting for these additional variables.

## Conclusions

Despite its limitations, this study makes several important contributions. Prior studies of ED travel patterns focused on specific geographic regions or insurance subgroups, or matched census data to hospital locations. A significant strength of our study is that we estimated the time/distance from a sample of the actual adult patient population (regardless of insurance status) of 50 hospital-based EDs across the country of varying size and locations, without restricting to a particular state, region, nationwide hospital system, or payer type.

The distance patients travel to EDs is relevant to healthcare policy, particularly access to care. Understanding how far and how willing patients are to travel is indicative of whether patient populations have adequate access to ED services. By showing that patients will travel an average of 6 min farther to avoid low-performing hospitals, we provide evidence that DTC patients likely do exercise some choice between EDs and that there are some market disincentives to hospitals providing poor-quality care, even in the ED setting. Understanding the degree to which ED patients have discretion to choose among EDs has implications for market share. When patients have such discretion, publicly reported ED quality measures can assist in the selection of higher-quality EDs, which could motivate all EDs to examine and improve their patients’ care. Understanding these issues will help policymakers to better define access to ED care and assist in directing quality improvement efforts.

## Supplementary Information

**Additional file 1. **

## Data Availability

The data that support the findings of this study may be available upon request from the Centers for Medicare & Medicaid Services via a data use agreement.

## Abbreviations

AHA: American Hospital Association
CAHPS: Consumer Assessment of Healthcare Providers and Systems
CI: Confidence Interval
CMS: Centers for Medicare & Medicaid Services
DTC: Discharged to the Community
ED: Emergency Department
EDPEC: Emergency Department Patient Experience of Care
ER: Emergency Room
HCAHPS: Hospital Consumer Assessment of Healthcare Providers and Systems
ref: Reference Category
SD: Standard Deviation
