# Supplementary material for: National travel distances for emergency care
Source: BMC Health Serv Res. 2022 Mar 24;22:388. doi: 10.1186/s12913-022-07743-7 (PMC8944092; doi:10.1186/s12913-022-07743-7)
Supplement: Supplementary file 1 — Additional file 1. [file 12913_2022_7743_MOESM1_ESM.docx]

ADDITIONAL FILE 1

Study Design and Participant/Hospital Selection

We conducted a nationwide study of adult ED patients who were discharged to home (as opposed to admitted to the hospital) from their ED visit.

To recruit hospitals to voluntarily participate in this study, we began by identifying hospitals with 14,000+ annual ED visits (in order to meet the study’s sampling requirements) using the 2013 American Hospital Association (AHA) database (15); children’s hospitals and other specialty hospitals were excluded. Note that freestanding emergency rooms (ER) were not recruited. All eligible hospitals were then placed into categories based on size and census region (e.g., hospitals in the Northeast with 50,000+ annual ED visits). We recruited a random proportionate stratified sample of eligible hospitals from each category in order to achieve a mix of hospital characteristics among our 50 participating hospitals. Hospitals did not receive monetary compensation for participation. Table 1 provides a comparison of characteristics of hospitals in our study with all hospitals in the 2015 AHA database (9) (excluding specialty hospitals and those without an emergency department).

Each participating hospital (or the hospital’s vendor) delivered patient discharges in six two-week sample batches, which included all discharges regardless of insurance status. Within each participating hospital, we extracted a simple random sample of eligible patients needed for the study, resulting in a total sample of 16,006 ED patients discharged between January 2016 and March 2016. Patient eligibility criteria paralleled those for the Hospital Consumer Assessment of Healthcare Providers and Systems (HCAHPS) Survey (7): patients were excluded if they were under age 18; had a primary diagnosis for mental health or substance use; were discharged to a hospital, nursing home, or skilled nursing facility; were transferred to another hospital; requested that they not be contacted; were court/law enforcement patients; had a foreign home address; died in the ED; or were admitted to the hospital following the ED visit. Patients were de-duplicated such that patients who were sampled for one ED visit would not be eligible for sampling based on additional ED visits occurring within the same month.

The sample frame included patient home addresses and telephone numbers. Sampled patients were given the Emergency Department Patient Experience of Care (EDPEC) Discharged to Community (DTC) Survey, developed by the Centers for Medicare & Medicaid Services (CMS) to measure patient experience of care in hospital-based emergency rooms. The survey is publicly available (1, 13, 14). Since the completion of this study, this survey received the Consumer Assessment of Healthcare Providers and Systems (CAHPS®) trademark in March 2020 and is now the ED CAHPS® Survey. The ED CAHPS Survey instrument and recommended guidelines describing survey administration and analysis are publicly available (14). For the purpose of this analysis, we used all eligible sampled patients from the study (including those that did not respond to the survey) with addresses in the US (excluding US Territories) that were within a 2-hour drive of the hospital-based ED they visited.

This study was approved by the Institutional Review Board at the study team’s institution. The content of the survey and additional details with regards to design and sampling, response rates, item scoring, and the patient population of the study have been described elsewhere (1).

Geocoding Process

Cleaned patient and hospital addresses were geocoded and used to calculate driving time and the Euclidean distance between patient and hospital addresses using ArcGIS Version 10.5 (8). Addresses were first loaded into an ArcGIS ArcMap document and geocoded using the NA_Local_Composite locator. The Intersect tool was run on the 2010 census block group shapefile and the patient point location shapefile to determine each patient’s census block group. The Generate Near Table function was used to calculate the Euclidean distance between the patient’s location and their hospital such that the patient location was the input dataset and the hospital location was the near dataset. An origin-destination cost matrix was created with the patient location as the origin and the hospital location as the destination to calculate the driving time (in minutes) between the patient’s location and their hospital with all traversing restrictions removed (time calculated based on quickest route using the road network without traffic allowing for travel on private roads, highways, etc.). The distance and driving time variables and the census block group data were combined using a Python script (16).

References

13. Weinick RM, Becker K, Parast L, Stucky BD, Elliott MN, Mathews M, et al. Emergency department patient experience of care survey: development and field test. Santa Monica: RAND Corporation; 2014.

14. Emergency Department CAHPS (ED CAHPS) Centers for Medicare and Medicaid Serivces. Available from: <https://www.cms.gov/Research-​Statistics-​Data-​and-​Systems/Research/CAHPS/ed.html. Accessed 9 Oct 2020>.

15. AHA annual survey database. American Hospital Association. 2013.

16. van Rossum G. Python tutorial, technical report CS-r9526, centrum voor Wiskunde en Informatica (CWI); 1995.
